# Chronic dietary oxalate nephropathy after intensive dietary weight loss regimen

**DOI:** 10.15171/jnp.2017.21

**Published:** 2017-02-05

**Authors:** Gebran Khneizer, Ahmad Al-Taee, Meher S Mallick, Bahar Bastani

**Affiliations:** ^1^Department of Internal Medicine, Saint Louis University School of Medicine, Saint Louis, Missouri, USA; ^2^Saint Louis Nephrology Associates, Saint Louis, Missouri, USA

**Keywords:** Oxalate nephropathy, Dietary hyperoxaluria, Acute kidney injury, Chronic kidney disease, Hyperoxaluria, Weight loss, Obesity

## Abstract

**Background:**

Hyperoxaluria has been associated with nephrolithiasis as well as acute and chronic kidney disease. We present a case of end stage renal failure caused by excessive dietary oxalate intake in a dietary weight loss regimen.

**Case Presentation:**

A 51-year-old Caucasian male with the past medical history of type 2 diabetes mellitus, gout, hypertension and morbid obesity was referred to the primary care clinic after being found pale and easily fatigued. The patient had lost 36 kg over a 7-month period by implementing exercise and intense dietary measures that included 6 meals of spinach, kale, berries, and nuts. Physical examination revealed a blood pressure of 188/93 mm Hg with sunken eyes and dry mucus membranes. Laboratory workup was notable for blood urea nitrogen of 122 mg/dL, creatinine of 12 mg/dL, and estimated glomerular filtration rate (eGFR) of 4.4 mL/min/1.73m2. Patient denied any history of renal disease or renal stones, or taking herbal products, non-steroidal anti-inflammatory drugs, antifreeze (ethylene glycol), or any type of "diet pills." Family history was unremarkable for any renal diseases. After failing intravenous fluid resuscitation, patient was started on maintenance hemodialysis. Abdominal imaging was consistent with chronic renal parenchymal disease with no evidence of nephrolithiasis. Renal biopsy revealed numerous polarized oxalate crystal deposition and diabetic nephropathy class IIA. At this point the patient was instructed to adopt a low oxalate diet. A 24-hour urine collection was remarkable for pH 4.7, citrate <50 mg, and oxalate 46 mg. Importantly, serum oxalate level was undetectable. Repeat renal biopsy 5 months later while patient was still on maintenance hemodialysis revealed persistence of extensive oxalate crystal deposition. Patient has been referred for evaluation for renal transplantation.

**Conclusions:**

Clinicians need to maintain a high index of suspicion for dietary hyperoxaluria as a potential etiology for acute or chronic kidney failure, particularly in patients pursuing intensive dietary weight loss intervention

**Implication for health policy/practice/research/medical education:**

Clinicians need to be familiar with dietary oxalate nephropathy in the setting of growing use of dietary weight loss regimens with high oxalate content.

## 1. Introduction

Hyperoxaluria, which is defined as excessive urinary oxalate excretion, has been associated with nephrolithiasis, and acute or chronic kidney injury. While there is ample literature on the link between hyperoxaluria and nephrolithiasis, there exists a paucity of reports on dietary oxalate association with renal disease (1). Hereby, we present a case of severe renal disease caused by excessive dietary oxalate intake.

## 2. Case Presentation

A 51-year-old Caucasian male with the past medical history of type 2 diabetes mellitus of 2 years duration, hyperuricemia and gout, hypertension, and morbid obesity decided to implement intense lifestyle modification including both dietary and exercise measures 7 months prior to presentation. He had been on a strict diet consisting of 6 meals of spinach, kale, berries, and nuts. In addition, he started a regular aerobic exercise regimen 5 times a week. With all these interventions, he was able to successfully lose about 36 kg.

His gym trainer noticed that he had become pale and was more easily fatigued at which point he was referred to a primary care clinic. Vital signs were notable for blood pressure of 188/93 mm Hg and weight of 145 kg. Physical examination revealed sunken eyes and dry mucus membranes. Laboratory work up was notable for blood urea nitrogen of 122 mg/dL, creatinine of 12 mg/dL, and estimated glomerular filtration rate (eGFR) of 4.4 mL/min/1.73m^2^. Patient was transferred to a nearby emergency department where he was started on intravenous fluids. Medications included daily aspirin, metoprolol and metformin. The patient denied any history of renal diseases or renal stone, or using tobacco, alcohol, ethylene glycol (antifreeze) or illicit drugs. He also denied taking any herbal products, non-steroidal anti-inflammatory drugs, or any type of “diet pills.” Family history was not remarkable for any renal or cardiovascular diseases. Patient was started on maintenance hemodialysis 3 times weekly.

Workup for renal failure included a renal ultrasound remarkable for kidneys 11-12 cm in length, increased bilateral parenchymal echogenicity compatible with chronic renal parenchymal disease, and no solid renal mass or hydronephrosis. Urine output throughout the course of the illness was around 1 to 2 L/d. Patient underwent a renal biopsy that revealed numerous polarized oxalate crystals in tubules and interstitium, interstitial inflammation, and mild mesangial expansion suggestive of diabetic nephropathy class IIA. Two out of 6 glomeruli showed global glomerulosclerosis with 30% tubular atrophy and 50% interstitial fibrosis. At this point the patient was instructed to adopt a low oxalate diet and to continue hemodialysis 3 times weekly.

Further workup to determine the etiology of oxalate nephropathy included abdominal imaging in the form of plain x-ray and computerized tomography (CT) scan which did not reveal any evidence of nephrolithiasis or nephrocalcinosis. A 24-hour urine collection for stone risk profile revealed: total urine volume of 1 L, pH 4.7, Ca 11 mg, Na 20 mEq, citrate <50 mg, oxalate 46 mg, uric acid 93 mg, phosphorous 580 mg, sulfate 13.4 mmol (reference range: 3.7-29 mmol/24 h), and creatinine 1240 mg. Importantly, serum oxalate level was undetectable.

**Figure 1:**
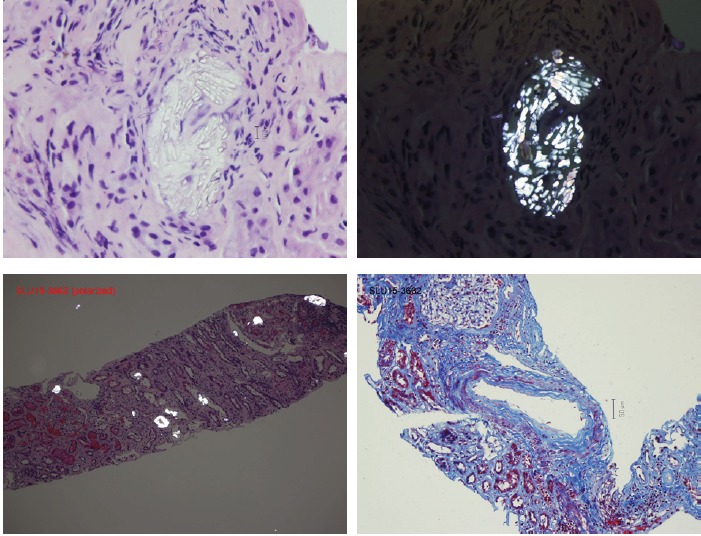
Upper left (H&E Stain) shows a tubule in renal cortex filled with oxalate crystals. Upper right shows the polarized picture of the same field. Lower left (polarized light) shows abundance of oxalate crystals in renal cortex. Lower right (Masson’s Trichrome Stain) shows the degree of interstitial fibrosis and tubular atrophy in the renal cortex.

A repeat renal biopsy 5 months later, while patient was still on maintenance hemodialysis, revealed mild mesangial expansion suggestive of diabetic nephropathy class IIA, and extensive oxalate crystal deposition in the tubules with associated interstitial inflammation and non-necrotizing granuloma. There was approximately 40% tubule atrophy and 70% interstitial fibrosis (Figure 1). Patient was referred for evaluation for renal transplantation.

## 3. Discussion

Hyperoxaluria develops as a consequence of either excessive endogenous oxalate production (primary hyperoxaluria) or excessive oxalate absorption from the gastrointestinal tract. The later can be associated with enteric hyperoxaluria from fat malabsorption, e.g., in patients with Roux-en-Y gastric bypass surgery (2), jejunoileal bypass surgery (3), Crohn’s disease (4), sprue (5) or taking medications such as orlistat that cause fat malabsorption (6). Other potential culprits for excessive oxalate absorption include either excessive dietary intake of oxalate or its substrate (vitamin C) or dietary deficiency of calcium or magnesium (1,7).

Diagnosis of dietary hyperoxaluria is based on the following criteria: First, one should rule out other potential causes of hyperoxaluria, i.e., primary hyperoxaluria (hereditary), and enteric hyperoxaluria (fat malabsorption). Second, documentation of excessive intake of products with high oxalate content. Third, normalization of hyperoxaluria after discontinuation of the excessive dietary oxalate intake (1). In the presented case, diagnosis of dietary hyperoxaluria was suggested by the patient’s excessive spinach and nuts intake. Primary hyperoxaluria was ruled out by undetectable serum oxalate level while patient was maintained on hemodialysis.

Dietary hyperoxaluria has been reported after ingestion of large amounts of food products with high oxalate content for medicinal, cosmetic or weight loss purposes. Most commonly implicated vegetables and fruits are peanuts (8), *Averrhoa bilimbi* (commonly known as bilimbi or cucumber tree) (9,10), celery, carrots, parsley, beets, and spinach (11,12).

Even though those food products are ubiquitous in many diets, there have been few reports of renal parenchymal disease secondary to dietary hyperoxaluria (1). Bakul et al have described a case series of 10 patients who developed acute renal failure after consumption of *A. bilimbi* fruit juice (9). Seven patients required hemodialysis while 3 cases recovered kidney function with conservative management. While their serum creatinine values at time of diagnosis ranged from 5.5 to 12.3 mg/dL, all 10 patients recovered within 2 to 6 weeks. Their serum creatinine levels at recovery varied between 0.8 and 2.1 mg/dL. Another report of 2 cases of acute oxalate nephropathy after ingestion of *A. bilimbi* juice has been described by Nair et al (10). While one of the cases was managed conservatively, the other underwent 4 sessions of hemodialysis along with a 3-day course of methylprednisolone. Both patients’ renal function recovered 3 weeks after presentation with serum creatinines at 1.4 and 2.1 mg/dL.

To the best of our knowledge the present case is the first report in the literature of severe irreversible renal failure due to dietary oxaluria from a weight reduction diet rich in oxalate (spinach and nuts). Our case is also unique in that unlike most of the reported cases of dietary oxalate nephropathy his renal function had progressed to end stage renal failure at the time of presentation.

Since the impact of dietary habits on nephrolithiasis has been well established (13), clinicians need to consider the role of dietary habits when evaluating patients with acute or chronic kidney diseases. ****

## 4. Conclusions

Clinicians need to maintain a high index of suspicion for dietary hyperoxaluria as a potential etiology of acute or chronic kidney diseases, particularly in patients pursuing intensive dietary weight loss intervention. Inquiring about dietary habits, as a part of history taking, is strongly advocated for the diagnosis of dietary hyperoxaluria.

## Acknowledgements

The authors would like to thank Dr. David Brink, the nephropathologist at Saint Louis University Hospital, for reviewing the pathology slides.

## Authors’ contribution

All authors contributed equally to the preparation of the case report.

## Conflicts of interest

The authors declare no conflict of interest.

## Funding/Support

There is no source of funding for this publication.
